# Insecticide Resistance Mechanisms in the Green Peach Aphid *Myzus persicae* (Hemiptera: Aphididae) I: A Transcriptomic Survey

**DOI:** 10.1371/journal.pone.0036366

**Published:** 2012-06-07

**Authors:** Andrea X. Silva, Georg Jander, Horacio Samaniego, John S Ramsey, Christian C. Figueroa

**Affiliations:** 1 Instituto de Ciencias Ambientales y Evolutivas, Facultad de Ciencias, Universidad Austral de Chile, Valdivia, Chile; 2 Boyce Thompson Institute for Plant Research, Ithaca, New York, United States of America; 3 Instituto de Silvicultura, Facultad de Ciencias Forestales y Recursos Naturales, Universidad Austral de Chile, Valdivia, Chile; 4 Center for Nonlinear Studies, Los Alamos National Laboratory, Los Alamos, New Mexico, United States of America; U. Kentucky, United States of America

## Abstract

**Background:**

Insecticide resistance is one of the best examples of rapid micro-evolution found in nature. Since the development of the first synthetic insecticide in 1939, humans have invested considerable effort to stay ahead of resistance phenotypes that repeatedly develop in insects. Aphids are a group of insects that have become global pests in agriculture and frequently exhibit insecticide resistance. The green peach aphid, *Myzus persicae*, has developed resistance to at least seventy different synthetic compounds, and different insecticide resistance mechanisms have been reported worldwide.

**Methodology/Principal Findings:**

To further characterize this resistance, we analyzed genome-wide transcriptional responses in three genotypes of *M. persicae*, each exhibiting different resistance mechanisms, in response to an anti-cholinesterase insecticide. The sensitive genotype (exhibiting no resistance mechanism) responded to the insecticide by up-regulating 183 genes primarily ones related to energy metabolism, detoxifying enzymes, proteins of extracellular transport, peptidases and cuticular proteins. The second genotype (resistant through a *kdr* sodium channel mutation), up-regulated 17 genes coding for detoxifying enzymes, peptidase and cuticular proteins. Finally, a multiply resistant genotype (carrying *kdr* and a modified acetylcholinesterase), up-regulated only 7 genes, appears not to require induced insecticide detoxification, and instead down-regulated many genes.

**Conclusions/Significance:**

This study suggests strongly that insecticide resistance in *M. persicae* is more complex that has been described, with the participation of a broad array of resistance mechanisms. The sensitive genotype exhibited the highest transcriptional plasticity, accounting for the wide range of potential adaptations to insecticides that this species can evolve. In contrast, the multiply resistant genotype exhibited a low transcriptional plasticity, even for the expression of genes encoding enzymes involved in insecticide detoxification. Our results emphasize the value of microarray studies to search for regulated genes in insects, but also highlights the many ways those different genotypes can assemble resistant phenotypes depending on the environmental pressure.

## Introduction

Insecticide resistance is one of the best examples of micro-evolution, or evolution occurring on an ecological time scale [1]–[3]. The study of insecticide resistance is important, both because it leads to a better understanding of evolutionary mechanisms operating in real time, and because of its economic relevance. The development of insecticide resistance in pest insects has been an increasing problem for agriculture, forestry and public health [4], [5]. Agricultural practices usually include the systematic application of a wide array of active compounds at variable dosages and frequencies, which represent a wide range of selective regimes. Therefore, identifying the molecular and genetic adaptations responsible for insecticide resistance will offer new opportunities for developing pest management strategies.

The study of insecticide resistance makes it possible to classify adaptations into three main mechanisms: (i) reduction of insecticide uptake, by reducing the permeability of insect cuticle [6]–[8], (ii) detoxification, through alteration in the levels or enzyme activities that degrade or sequester insecticides [1], [7], [9]–[11] and, (iii) insensitivity due to point mutations in genes encoding for proteins that are the target site of insecticides [12]–[14]. Functional genomics tools have recently been used to disentangle the genetic basis of pesticide resistance in arthropods [15]–[23]. Such studies have shown that insecticide resistance is more complex than previously thought, being mediated by multigenic systems that involve large parts of the insect genomes [10], [18], [24].

Aphids (Hemiptera: Aphididae) are widely distributed herbivorous insects accounting for more than 4,300 described species [25]–[27]. Approximately 100 aphid species have successfully exploited agro-ecosystems to become economically important pests, of whom ∼20 have developed at least one known insecticide resistance mechanism [28], [29]. The peach green aphid, *Myzus persicae*, of Palearctic origin, is a cosmopolitan aphid species responsible of important economic losses [26], [30], [31]. Is a highly polyphagous, feeding on more than 50 plant families [30], [32], causing losses to agroindustrial crops (including potato, sugar beet and tobacco), horticultural crops (including plants of Brassicaceae, Solanaceae and Cucurbitaceae families) and stone fruits (peach, apricot, and cherry, among others). *M. persicae* was introduced into Chile with crop plant species [33], and is presently categorized as one of the three most important agricultural pests in this country [34].

*M. persicae* exhibits a striking capacity for rapid adaptation to insecticides, developing resistance to more active compounds than any other known insect [26], [35]. Six distinct insecticide resistance mechanisms mediating different levels of insensitivity, have been described for the species: (i) Modified acetylcholinesterase (MACE), which confers resistance to organophosphates and carbamate insecticides [36]–[39], (ii) *kdr* and *super kdr* mutations in a voltage-gated sodium channel, which is the target of pyrethroids and organochlorines [40]–[42], (iii) the mutation of the GABA receptor, *rdl,* which is target of organochlorines of the cyclodiene type [43], [44], (iv) the recently described mutation of a key residue in the loop D region of a nAChR b1 subunit [45], (v) the over-production of esterases E4 or FE4 confers resistance to organophosphates, pyrethroids and to a lesser extent carbamates [46]–[51], and (vi) the recently described over-production of a cytochrome P450 confers resistance to neonicotinoids [16], [45], [52].

In Chile, *M. persica*e has been chemically controlled by the application of almost all classes of insecticides, including neonicotinoids, pyrethroids, organophosphates and carbamates. Pirimicarb, an anti-cholinesterase insecticide, is the most frequently used since the last five years. However, little is known about the insecticide resistance mechanisms of *M. persicae* in Chile. For instance, esterase-mediated resistance (E4/FE4) has been found in *M. persicae* on sugar beet crops (*Beta vulgaris*), with phenotypes ranging from R1 (moderately resistant) to R3 (highly resistant) [53]–[55] In contrast, on tobacco (*Nicotiana tabacum*) only a single and widely distributed clone has been reported, which exhibits a R1 phenotype for esterases and susceptibility at the site of the *kdr* mutation [56].

Transcriptomics is an extremely useful approach for the identification of new genes and gene functions related to insecticide resistance [57]. DNA microarrays, one of the most powerful and versatile transcriptomic techniques, make it possible to compare expression profiles for hundreds or thousands of genes simultaneously, thereby linking the study of static genomes to dynamic proteomes [58]. Although the genome of *M. persicae* has not been sequenced yet, genomic resources are available for this species [24], [59]. Recently, two studies have targeted the identification of insecticide resistance mechanisms in *M. persicae* using genomic resources in an integrated fashion [16], [45]. In both cases the focus was on discovering the mechanisms responsible for the neonicotinoid resistance, comparing patterns of gene expression between susceptible and resistant aphid clones [16], [45]. Following this methodology, one can identify new genes involved in insecticide resistance in populations, but it is not possible to detect the full potential of a species to evolve in response to insecticides.

In the current study, we took advantage of the recent advances in aphid genomics to examine the transcriptional responses in three genotypes of *M. persicae* exposed to pirimicarb at the whole-genome level. This approach allowed the comparison of the expression profiles in genotypes carrying different resistance mutations, thereby identifying new genes and mechanisms that are the target of selection.

## Results

### Insecticide Resistance Characterization

Thirty-two *M. persicae* genotypes were evaluated constitutive carboxylesterase activity (EST activity), which is indicative of the number of copies for E4/FE4 carboxylesterase genes [60]. EST activity was low for the 32 genotypes evaluated. Indeed, all genotype assayed were “susceptible” according to the classification of Devonshire et al. (1992) [61]. However, broad-sense heritability of EST activity was significant (H^2^ = 0.61; F_31,274_ = 15.8, P<0.0001), indicating a larger variation among than within genotypes, which validates the use of this variable in the selection of experimental lineages.

By characterizing the genetic makeup of insecticide resistance mutations (IRM), the 32 genotypes were grouped into three categories. Twenty-one genotypes did not carry any IRM and were labeled as sensitive (i.e. S genotypes). From this group, genotype 13A (hereafter S) exhibited the lowest level of EST activity and was selected for microarray experiments. Nine genotypes were heterozygous for *kdr*, carrying no MACE or *super-kdr* mutations, and were labeled as simple resistant (i.e. SR genotypes). From this group, genotype 26A (hereafter SR) was selected due to its intermediate EST activity. Finally, two genotypes were heterozygous for both *kdr* and MACE mutations and were labeled as multiple resistant (i.e. MR genotypes). From this group, genotype 16A (hereafter MR) was chosen due to its higher levels of EST activity. No other IRM combinations were found.

We found a significant link between the genetic constitution for IRM and the susceptibility of genotypes to insecticide, estimated from insecticide tolerance bioassays. The genotype S showed the lowest lethal dose values for pirimicarb, which results in increased susceptibility (LC_50_ = 9.27 ppm±0.13 EE), followed by genotype SR (LC_50_ = 11.44 ppm±0.22 EE); and genotype MR (LC_50_ = 407.45 ppm±0.13 EE). Descriptive characterizations of the three genotypes selected are presented in Table 1.

**Table 1:** Characterization of the *Myzus persicae* genotypes selected for microarray experiments.

Genotype	MACE	*kdr*	*s.kdr*	EST activity*	LC_50_ pirimicarb (CI 95%)
S	SS	SS	SS	0.150±0.03	9.27 (7.2–11.8)
SR	SS	SR	SS	0.207±0.01	11.44 (9.4–14.1)
MR	SR	SR	SS	0.291±0.02	407 (153–3965)

* (U aphid-equiv. ^−1^) ± SE.

### Microarray Experiments

**Figure 1:**
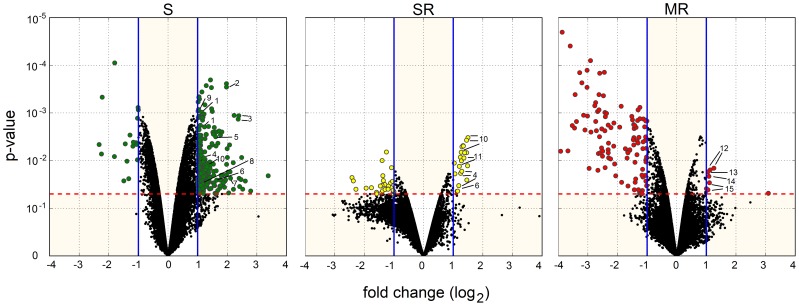
Transcriptional responses in three *Myzus persicae* genotypes (S, SR and MR) subjected to a pirimicarb. Volcano plots for each genotype show the log_2_ fold change (x axis) and the statistical significance (*y* axis) between the controls and treatments. Vertical lines indicate 2-fold expression difference in either direction (−1>log2FC>1). Horizontal line indicates significance threshold (P<0.05). Statistical analysis is based on a Bayesian inference using a lineal model, and reflects both biological and technical replications. Genes showing both 2-fold differential expression and a significant P value are colored. Not all labels appear in the S, SR and MR volcano plot in order to preserve readability (see Table 2 and supporting material for a full listing of significantly over-expressed genes). Gene abbreviations: 1, *glutathione s-transferase*; 2, *cytochrome p450 family CYP6CYP3*; 3, *carboxylesterase type FE4*; 4, *cathepsin b*; 5, *cytochrome p450 family CYP6*; 6, *cuticle protein*; 7, *salivary peptide*; 8, *ABC transporter*; 9, *glucose transporter*; 10, *cytochrome p450*; 11, *heat shock protein 70*; 12, *heterotrimeric guanine nucleotide-binding protein*; 13, *histone h3 methyltransferase*, 14, *eukaryotic initiation factor*; 15, *unknown protein*.

Microarray experiments were performed in order to study the transcriptome responses in three *M. persicae* genotypes (S, SR and MR) subjected to a dose of pirimicarb. Microarray analysis detected a high variation in transcriptional responses among genotypes. Global gene expression changes are shown in Figure 1 in the form of volcano plots, with threshold of 2-fold-change and a significance threshold of p<0.05. Thus, 183, 17 and 7 genes were significantly up-regulated in S, SR and RM genotypes, respectively. (see Table S1 for the full list of up-regulated genes). Interestingly, the number of down-regulated genes was inverse to the number of up-regulated genes in each genotype. Thus, 17, 28 and 78 genes were significantly down-regulated in S, SR and RM genotypes, respectively. Of the 183 up-regulated genes found in S genotype, 151 had known functions and 51 are potential candidates for being involved in insecticide resistance, including genes encoding for *abc* transporters, heat shock proteins, cathepsins, cuticle proteins, cytochrome P450s, a carboxylesterase E4/FE4 and glutathione-S-transferases, among others (see some of these genes in Table 2). Of the 17 up-regulated genes in the SR genotype, 12 have known functions and are potentially involved in insecticide resistance, including heat shock proteins, cathepsins, cuticle proteins and cytochrome P450s (Table 3). Finally, of the 7 up-regulated genes in the MR genotype, 3 have unknown functions while the other 4 genes included a histone h3 methyltransferase and a guanine nucleotide-binding protein (Table 4).

**Table 2:** Selected genes identified by microarray as significantly up-regulated in S (sensitive) genotype in response to pirimicarb.

Description	Hit Description	Log_2_ FC	Contig ID	Probe name
heat shock protein 70	gi|193647903|ref|XP_001945786.1	2,.9	6029	M_persicae6029a
heat shock protein 70	gi|193647903|ref|XP_001945786.1|	2.20	8669	M_persicae8669a
heat shock protein 70	gi|193688192|ref|XP_001951386.1|	1.03	15349	M_persicae15349a
carboxylesterase esterase fe4	gi|544256|sp|P35502.1|	2.39	9215	M_persicae9215a
carboxylesterase esterase E4	gi|544255|sp|P35501.1|	2.30	720	M_persicae720a/b
carboxylesterase esterase E4	gi|544255|sp|P35501.1|	1.42	4586	M_persicae4586a
Esterase	gi|544255|sp|P35501.1|	2.04	3118	M_persicae3118a/b
glutathione s-transferase	gi|193636685|ref|XP_001946604.1|	1.13	1196	M_persicae1196a
glutathione s-transferase	gi|193636685|ref|XP_001946604.1|	1.09	4744	M_persicae4744a
cytochrome p450 cyp6ax1	gi|193598913|ref|XP_001943150.1|	1.05	3931	M_persicae3931b
cytochrome p450 cyp6ax1	gi|193671582|ref|XP_001952450.1|	1.05	6957	M_persicae6957a
cytochrome p450 cyp6ax1	gi|193657143|ref|XP_001948488.1|	1.61	5173	M_persicae5173a
cytochrome p450 cyp6ax1	gi|193657143|ref|XP_001948488.1|	1.97	497	M_persicae497a/b
cytochrome p450	gi|193599086|ref|XP_001945361.1|	1.04	1528	M_persicae1528b
cytochrome p450 cyp6ax1	gi|193657145|ref|XP_001948581.1|	1.40	3798	M_persicae3798a/b
cytochrome p450 cyp6ax1	gi|193587097|ref|XP_001948421.1|	1.29	9095	M_persicae9095a
cytochrome p450 cyp6ax1	gi|193657145|ref|XP_001948581.1|	1.23	9584	M_persicae9584a
cytochrome p450 cyp6ax1	gi|193657145|ref|XP_001948581.1|	1.73	3799	M_persicae3799a
cytochrome p450 cyp6ax1	gi|193657143|ref|XP_001948488.1|	1.14	749	M_persicae749a/b
aldehyde dehydrogenase	gi|193617714|ref|XP_001949972.1|	1.37	2450	M_persicae2450b
cathepsin b–n	gi|51947600|gb|AAU14266.1|	1.49	256	M_persicae256a/b
cathepsin b–n	gi|51947600|gb|AAU14266.1|	1.26	254	M_persicae254a/b
cathepsin b	gi|161343867|tpg|DAA06114.1|	1.10	3004	M_persicae3004a
cathepsin b	gi|161343867|tpg|DAA06114.1|	1.09	3002	M_persicae3002b
cathepsin b–n	gi|193654855|ref|XP_001943173.1|	1.04	6594	M_persicae6594a
abc transporter	gi|193664711|ref|XP_001950287.1|	1.15	1560	M_persicae1560a/b
abc transporter	gi|193636433|ref|XP_001950956.1|	1.14	7913	M_persicae7913a
abc transporter	gi|193664711|ref|XP_001950287.1|	1.19	2478	M_persicae2478b

**Table 3:** Selected genes identified by microarray as significantly up-regulated in SR (simple resistant) genotype in response to pirimicarb.

Description	Hit Description	Log_2_ FC	Contig ID	Probe name
heat shock protein 70	gi|193647903|ref|XP_001945786.1|	1.36	6029	M_persicae6029a
heat shock protein 70	gi|193647903|ref|XP_001945786.1|	1.21	8669	M_persicae8669a
cytochrome p450	gi|193713785|ref|XP_001947768.1|	1.47	2519	M_persicae2519a/b
cytochrome p450	gi|193657315|ref|XP_001944487.1|	1.20	1504	M_persicae1504a/b
cathepsin b	gi|161343867|tpg|DAA06114.1|	1.06	3002	M_persicae3002b
cathepsin b	gi|51947600|gb|AAU14266.1|	1.20	256	M_persicae256a/b
cathepsin b	gi|161343867|tpg|DAA06114.1|	0.99	6891	M_persicae6891a
cuticular protein	gi|240848841|ref|NP_001155592.1|	1.13	10027	M_persicae10027a
cuticular protein	gi|193647875|ref|XP_001945170.1|	1.00	4497	M_persicae4497a
nonstructural protein ns-1	gi|33235700|ref|NP_874376.1|	1.26	3321	M_persicae3321a/b
protoheme ixfarnesyltransferase	gi|15617066|ref|NP_240279.1|	1.46	9124	M_persicae9124a
zinc mym domain	gi|193704454|ref|XP_001951785.1|	1.32	2558	M_persicae2558a/b

**Table 4:** Selected genes identified by microarray as significantly up-regulated in MR (multiple resistant) genotype in response to pirimicarb.

Description	Hit Description	Log_2_ FC	Contig ID	Probe name
yellow protein	gi|193683309|ref|XP_001945133.1|	3.09	6351	M_persicae6351a
guanine nucleotide-binding proteinsubunit beta 1	gi|193596402|ref|XP_001947878.1|	1.19	1180	M_persicae1180a/b
histone h3 methyltransferase	gi|193683706|ref|XP_001947040.1|	1.06	6961	M_persicae6961a
eukaryotic translation initiation factor 4 3	gi|193657071|ref|XP_001945066.1|	1.09	2227	M_persicae2227b

**Figure 2:**
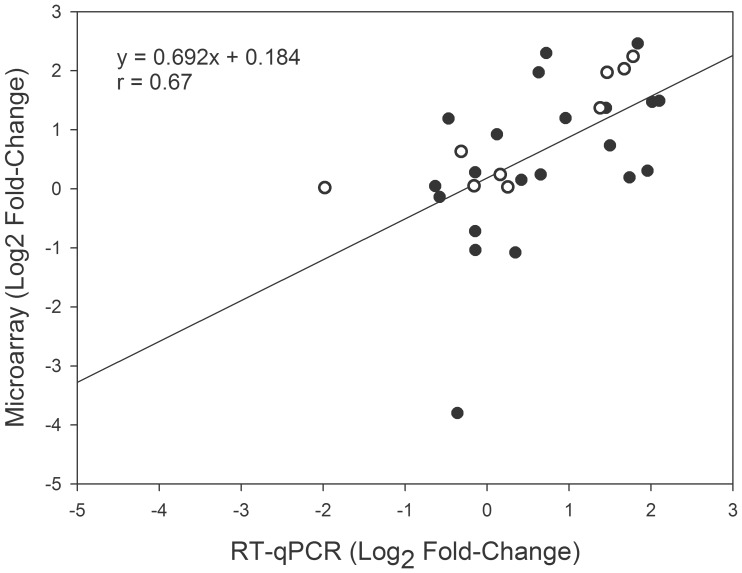
Correlation of gene expression changes measured using DNA microarray analysis and quantitative reverse transcription PCR (RT-qPCR). The average log2 fold-change values were used, and each point represents the gene expression in a genotype. Open circles correspond to expression using in RT-qPCR the same RNA samples as were used for microarray experiments. Black circles correspond to expression in RT-qPCR experiments using RNA that was obtained from new biological replicates. Spearman correlation coefficient (r) is shown in the graph.

In order to validate the microarray profiles, the transcriptional changes for seven up-regulated genes were studied by RT-qPCR in all the three genotypes, using RNA obtained from new biological replicates. Additionally, transcriptional profiles of three differentially expressed genes were validated using the same RNA samples used for microarray experiments. Comparisons of gene expression between the two techniques are shown in Figure 2 (r = 0.67; P<0.01; Spearman correlation coefficient) and gene expression results for both methodologies are listed in Table S2.

### Annotation and Gene Ontology Analysis

**Figure 3:**
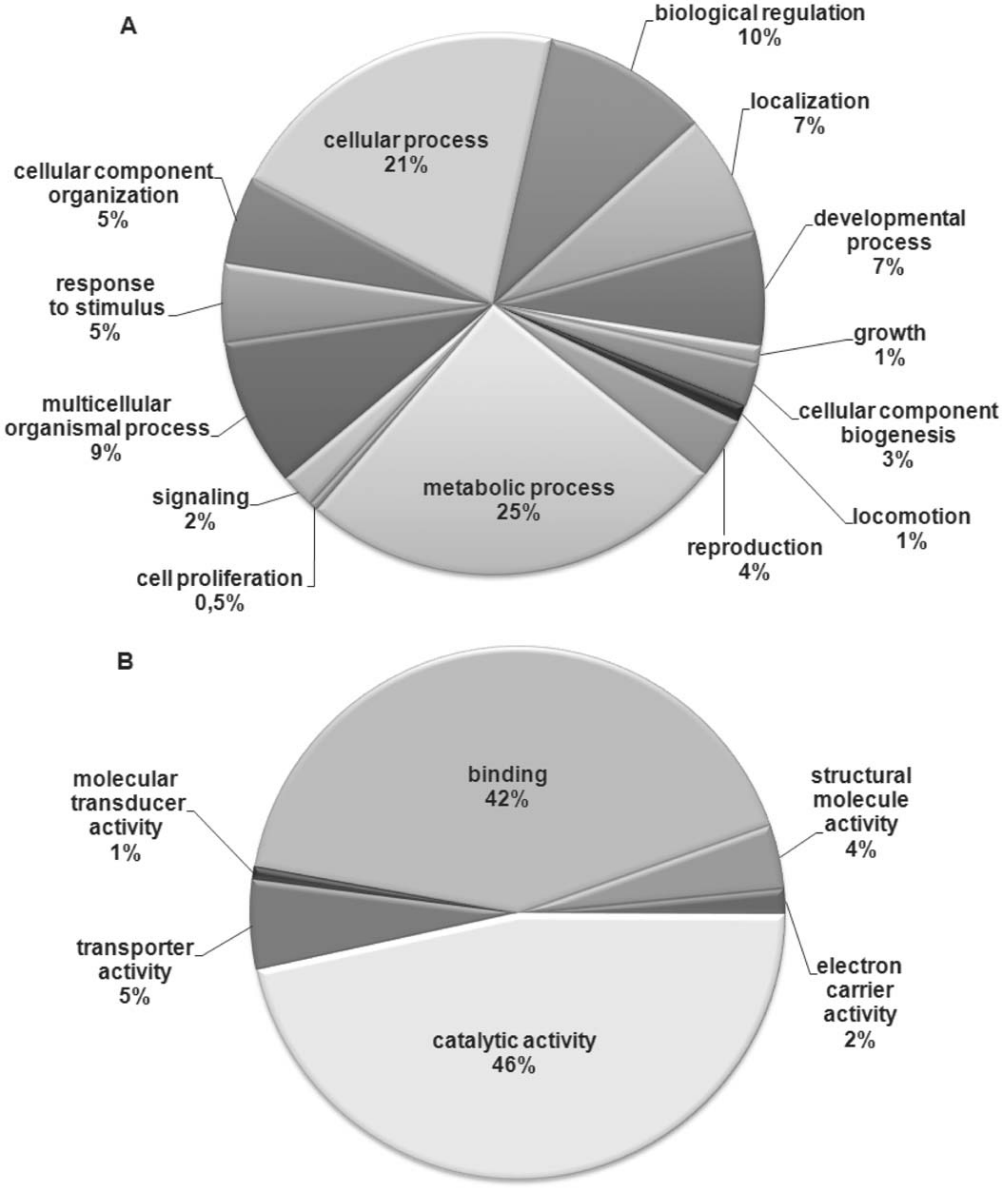
Distribution of GO IDs at the 2^nd^ level. Based on their participation in biological processes (A) and molecular functions (B) of up-regulated ESTs (putative proteins) in a sensitive genotype (S) of *Myzus persicae* treated with pirimicarb. Out of 97 annotated EST sequences, 69 presented GO IDs for biological processes and 88 for molecular functions.

A total of 97 sequences of 183 up-regulated genes in the S genotype were annotated. Gene Ontology (GO) graphs were constructed using percentages of 2^nd^ level GO terms and presented in Figure 3 under biological processes (BP) and molecular functions (MF). GO analysis revealed the participation of 69 putative proteins in 14 BP (Figure 3A). Among them, metabolic processes were the most represented with 49 gene products (25%) involved in primary metabolic processes (protein localization, carbohydrate and lipid biosynthetic and catabolic process, ATP and nucleotide biosynthetic process), cellular metabolic process (including the generation of precursors metabolites and energy) and oxidation reduction processes among others. The second largest represented group corresponded to putative proteins encoded by 40 genes (21%) and involved in cellular processes such as organelle organization, actin filament-based processes, microtubule-based processes, cell division, cytoplasm organization and cell communication. Under the category of molecular functions (MF), 88 gene products were involved in 6 different activities (some in more than one category) (Figure 3B). Most sequences (60 gene products) were related to catalytic activity; among them, the 44% corresponded to hydrolase activity (GO terms associated with *esterase* and *cathepsins*), 26% to transferase activity (GO terms associated with *glutathione-S-transferase*) and 16% to oxidoreductase activity (GO terms associated with *cytochrome P450s*).

**Figure 4:**
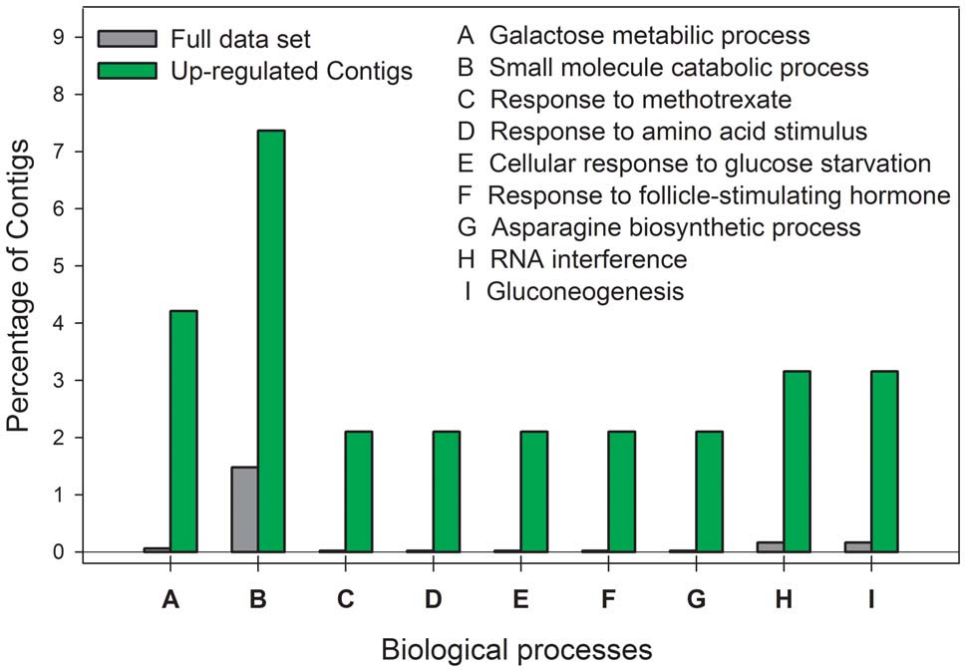
Biological processes over-represented in the sensitive genotype (S) after an Enrichment Analysis. The bars show the percentage of contigs associated with each GO term. The dark gray bars show the percentage of contigs associated with each GO term considering the full microarray data set. Green bars show the percentage of contigs associated with each GO terms, but only in the up-regulated date set

**Figure 5:**
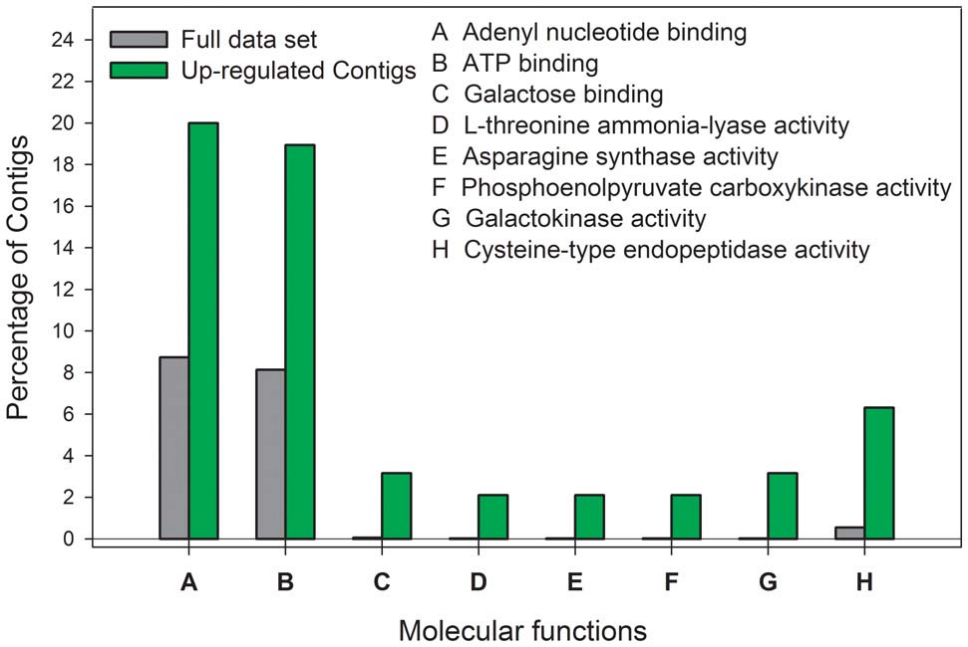
Molecular functions over-represented in the sensitive genotype (S) after an Enrichment Analysis. The bars show the percentage of contigs associated with each GO term. The dark gray show the percentage of contigs associated with each GO term considering the full microarray data set. Green bars show the percentage of contigs associated with each GO term but only in the up-regulated date set.

An enrichment analysis (EA) revealed that BP and MF were significantly over-represented among the up-regulated sequences in the genotype S with respect to all sequences in the microarray. The analysis within the BP category revealed that gluconeogenesis, small molecule catabolism, cellular response to glucose starvation, response to amino acid stimulus, among others, were significantly over-represented (Figure 4). The analysis within the MF category showed an over-representation of catalytic activity including peptidase, hydrolase, kinase, and lyase activities (Figure 5).

### Transcriptional Levels for Specific Genes

We evaluated the transcriptional expression for seven genes that were found up-regulated in the three studied genotypes, 20 and 30 hours after the application of pirimicarb.

**Figure 6:**
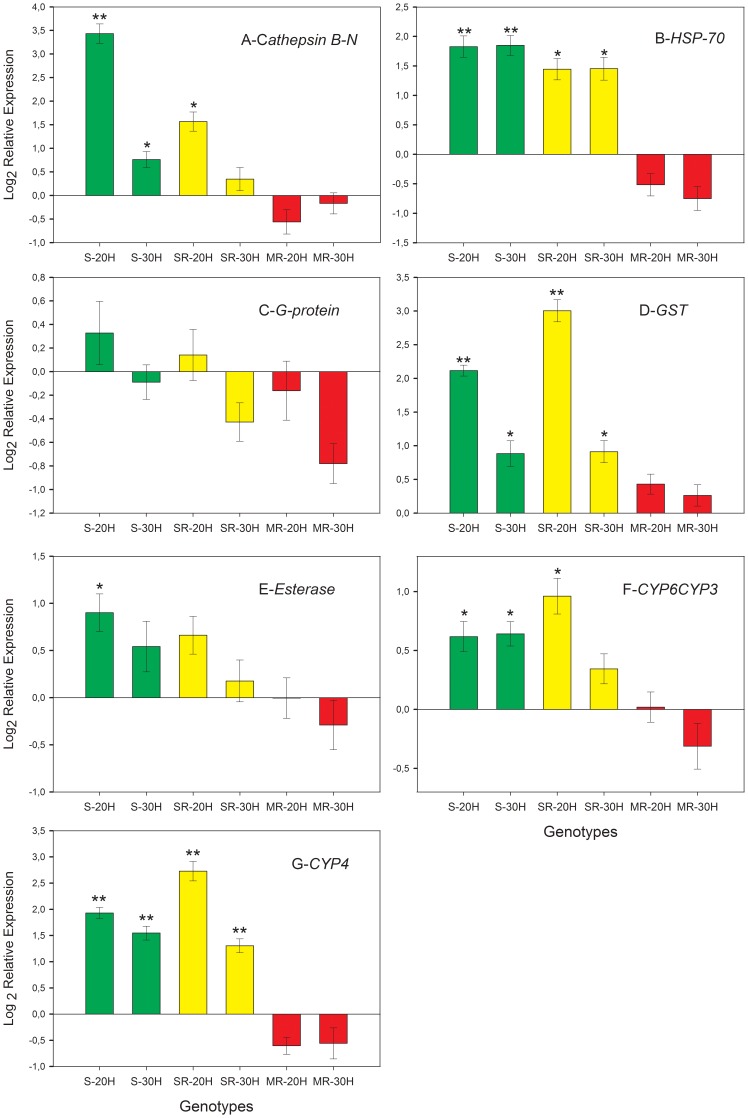
Quantification of relative expression in different genotypes of *Myzus persicae* exposed to pirimicarb. Graphs represent the relative mRNA expression in aphids sprayed with pirimicarb in comparison to control (water). Data were normalized for interclonal variation using GADPH expression levels. Green bars correspond to the genotype S (sensitive), Yellow corresponds to the genotype SR (simple resistant) and red bars correspond to the genotype MR (multiple resistant). Same color bars represent the time after insecticide spraying, with left bar = 20 hours and right bar = 30 hours. Data are shown as mean ± SE of two independent experiments, with three technical replicates in each case. *p<0.05 and **p<0.01 indicate a significant difference compared to 1, which was used as a reference value for no change in gene expression, using a *t-test*. Gene abbreviations: (A) *cathepsin B–N*, *cathepsin B clade N*; (B) *HSP-70, heat shock protein 70*; (C) *G protein*, *Heterotrimeric guanine nucleotide-binding protein*; (D) *GST*, *glutathione S-transferase*; (E) *Esterase*, *carboxylesterase type E4/FE4*; (F) *CYP6CYP3*, *cytochrome p450 family CYP6CYP3*; (G) *CYP4*, *cytochrome p450 family CYP4*.

The *Cathepsin B* gene showed a significant up-regulation in the S and SR genotypes at 20 hours after insecticide application, while at 30 hours up-regulation remained significant only in the S genotype. Genotype MR showed no evidence of regulation for this gene (Figure 6A). The *Heat Shock Protein 70* gene showed a significant up-regulation in S and SR genotypes at 20 and 30 hours after the application of the insecticide. In contrast, the MR genotype showed a down-regulation for this gene (Figure 6B). The *Heterotrimeric G protein* gene did not show a significantly different transcription between treatments in any of the studied genotypes (Figure 6C). In this case, we found transcriptional differences between the results obtained by the microarray analysis compared to the RT-qPCR, which can be explained by intra-clonal variation (see Discussion). The *Glutathione-S-transferase* gene showed a significantly higher transcription in S and SR genotypes at 20 and 30 hours after the application of insecticide (Figure 6D), while no changes were detected in the MR genotype. The *Esterase* gene only showed a significant up-regulation at 20 hours after application of insecticide in the S genotype (Figure 6E), while all other genotypes were unaffected. Two genes of the *Cytochrome P450* gene family were assessed (*CYP6CY3* and *CYP4*). The genotypes S and SR showed an up-regulation for both genes at 20 and 30 hours after application of insecticide (Figure 6E and 6G), while the genotype MR showed no changes.

## Discussion

Insecticide resistance is a textbook example of rapid evolution occurring in front of our eyes. The aphid *M. persicae* holds the world record of insecticide resistance mechanisms, showing resistance to at least seventy different synthetic compounds [62]. This fact alone makes this species an exceptional model for studying the genes and mechanisms that are the target of insecticide selection. In this study, different field-derived genotypes were characterized for three well-described insecticide resistance mechanisms (EST activity, and *kdr* and MACE mutations). This characterization, together with pirimicarb tolerance bioassays, allowed us to select three genotypes: sensitive (S), resistant only by a *kdr* mutation (SR) and resistant by *kdr* and MACE mutations (MR).

The transcriptomic responses of these three genotypes exposed to pirimicarb, provided evidence of a high variation in transcriptional plasticity among genotypes. Although slight discrepancies were observed for the transcriptional profiles given by the microarray and RT-qPCR approaches, these differences can be explained by the use of different biological replicates for each technique, and because aphids are especially known to show intra-clonal variation [63], [64]. In the microarray experiments, the number of up-regulated genes was inversely correlated to insecticide resistance mechanisms. To better understand the observed responses, it is necessary to emphasize that pirimicarb is an anti-cholinesterase insecticide, acting by inhibiting the enzyme acetylcholinesterase [65]. Hence, the MACE mutation (carried only by the MR genotype) confers specific resistance to this class of insecticides.

The different transcriptomic responses found between S and SR genotypes may be associated with *kdr* mutation, which causes insecticide insensibility in the sodium channel. The *kdr* mutation should have no effect on resistance to an anti-cholinersterase insecticide. However, epistasis with another insecticide resistance mechanism could be invoked here, as it has been reported in *Culex* and *Aedes* mosquitoes [66], [67]. In these cases, epistasis occurs between the *kdr* mutation and an enhanced detoxification by cytochrome P450 monooxygenases, while in *M. persicae* a strong linkage disequilibrium between this mutation and insecticide resistance mediated by esterases has been found [68]–[70]. However, we cannot exclude the possibility that the up-regulation shown by the S genotype relative to SR and MR is actually a general stress response due to the inhibition of cholinesterase by the pirimicarb insecticide, rather than a specific resistance response.

### General Metabolic Responses to Insecticides

Among the 183 up-regulated genes found in the S genotype after insecticide application, the most interesting observation was the unusual activation of energy metabolism. It was evident the up-regulation of several key enzymes in metabolic pathways affecting glycolysis, gluconeogenesis, the Krebs cycle, galactose, lipids, and amino acid metabolism. This was consistent with the fact that insecticide resistance is usually associated with higher demands of energy in other insect species [7], [71], [72]. In other words, when facing insecticides, aphids of the S genotype experience an increase of general metabolism (both aerobic and anaerobic) that is accompanied by the mobilization of energy stores (glycogen and fats). Our findings suggest that gene expression that promotes mobilization of energy may somehow mitigate the costs of insecticide action (e.g. muscle contractions and insecticide detoxification), even 24 hours after the application of pirimicarb. In contrast, in the SR genotype (17 up-regulated genes, carrying *kdr* mutation), only detoxifying enzymes were found to be up-regulated, with no evidence for the activation of energetic metabolism and muscle contraction. Interestingly, in the MR genotype (7 genes up-regulated; carrying MACE and *kdr* mutations), neither metabolic nor detoxifying genes were found to be up-regulated, which strongly suggests that resistance is also related to insecticide entry into the haemolymph.

### Detoxification Genes

Transcriptomic responses are discussed separately for genotypes that carry (MR) or do not carry (S and SR) the MACE mutation. Four types of catalytic reactions are known to be involved during insecticide detoxification; hydrolysis, oxidation, reduction and conjugation [65], [73]. Hence, genes coding for enzymes participating in those reactions are putatively involved in resistance. Among the up-regulated unigenes in the S genotype, hydrolase activity was significantly over-represented with 27 unigenes, four of them encoding for carboxylesterase FE4 and its closely variant E4 (contigs ID 3118, 9215, 720, and 4586). Previous studies have reported the constitutive up-regulation (up to 290-fold) of these same contigs in a *M. persicae* genotype resistant to neonicotinoids [16], [45]. Those contigs showed a good match (E-value ranging between 0 to 3E-172) with *E4* gene from clone 794J, which has been characterized as “extremely resistant” (R3) because of the *E4* gene amplification involving about 80 copies [47], [74].

The cytochrome P450s (*CYP* genes) catalyze the oxidation of insecticides, being the only metabolic system involved in resistance to all classes of insecticides [12], [14], [75]–[77]. Eleven P450s unigenes (contigs ID 497, 5173, 1730, 3931, 6957, 1528, 3799, 3798, 9095, 9584, 749) and two (contigs ID 2519 y 1504) were found to be up-regulated in the S and RS genotypes, respectively. It has been estimated that *M. persicae* has over 150 *CYP* genes, approximately 40% more than *Acyrthosiphon pisum* (the only aphid species with a whole genome sequence available) [78]. Why has the expansion of this gene family been favored during the evolution of *M. persicae*? The number of *CYP* genes in *M. persicae* has perhaps granted a range of functional diversity to this aphid, thus promoting insecticide resistance by different metabolic pathways. Indeed, three of the up-regulated contigs found in the S genotype (contigs ID 497, 5173, 749, corresponding to *CYPCY6* gene) also have been shown to be constitutively up-regulated (9 to 22 fold) due to gene amplification in a neonicotinoid resistant *M. persicae* genotype [16], [45].

The consistency in the up-regulation observed for *E4* and *CYPCY6* genes between the sensitive genotypes studied here, and the constitutive up-regulation by gene duplication in other resistant genotypes, is in agreement with the model for the resistance changes proposed before [1], [79], [80]. In the absence of resistance mutations or when the frequency of resistant alleles is low in populations, most individuals are susceptible, responding to insecticides by the up-regulation of some specific genes. When the selective agent (insecticide) acts within the limits of tolerance of the initial population, a marginal increase in tolerance has been observed, thus promoting selection of different traits with a low but accumulative effect on resistance (i.e., polygenic resistance). Given the massive application of insecticides in agricultural fields, it would be expected that selection for resistance has operated at the extremes of the phenotypic distribution for resistance. Thus, large-effect mutations accumulate, the retention of duplication events for those genes is promoted, and the up-regulation becomes constitutive. This scenario highlights the importance of analyzing the gene expression in susceptible genotypes when one is searching targets of selection.

Cytochrome P450s have traditionally been considered as the only enzymes to oxidize insecticides in insects [11], [65]. However, an aldehyde dehydrogenase (contig ID 2450) was also shown to be up-regulated (2.6-fold) in the S genotype. In mammals, aldehyde dehydrogenases have been described as important enzymes during the detoxification of xenobiotics [81], [82], and have recently been suggested to participate in the detoxification of pyrethroid in insects [22], [83]. Hence, the up-regulation of contig 2450 found in this study provides new evidence for understanding its detoxifying role as part of the insecticide metabolism in insects.

Regarding carbamates metabolism, most literature involves the action of glutathione S transferases (GSTs) in phase II of carbamate detoxification. GSTs are able to conjugate glutathione with phase I metabolites, converting them into non-reactive water-soluble conjugates [11], [65]. Curiously, it has not been possible so far to find a sole empirical study in insects linking GST with carbamate metabolism, whereas other works have characterized the role of GSTs in organophosphate, organochlorines and pyrethroid detoxification (see [12], [14]). GSTs also play an important role in cell protection, participating indirectly in insecticide resistance by reducing the oxidative damage caused by insecticides [9], [84], [85]. In the S genotype, two GST unigenes were found to be up-regulated (contigs ID 1196, 4744), but our experimental design does not allow us to anticipate any mechanism behind this up-regulation.

UDP-glucuronosyltransferases (UGTs) are another group of conjugative enzymes involved in phase II detoxification. In DDT-resistant strains of *Drosophila*, for example, UGT is constitutively expressed [18], whereas in *Anopheles* it was shown to be up-regulated after permethrin application [22]. We identified a UGT transcript (contig ID 8298) that is up-regulated in the S genotype, thus extending the range of potential UGT efficacy to carbamate detoxification. Although the SR genotype did not provide evidence of the participation of this enzyme, the transcriptomic response was obtained after 24 hours of pirimicarb treatment (based on a preliminary LC_50_ experiments in sensitive genotypes), and we cannot exclude the possibility that other genes could be significantly up-regulated at a different time. Indeed, our RT-qPCR profiles of the SR genotype clearly showed an eight-fold up-regulation of contig 1196 encoding GST after 20 hours of insecticide treatment.

### Transcripts Coding for Other Potentially Relevant Proteins

Two unigenes (contigs ID 8669 and 6029) encoding heat shock proteins 70 were found up-regulated in the S and SR genotypes. Proteins of the HSP70 family are particularly well studied and correspond to one of the first known mechanisms in stress responses [86]. Insecticide resistance is also commonly associated with the expression of HSP70 [87], [88]. Thus, our results showing a HSP70 induction in S and SR genotypes, would give evidences that insects are trying to restore cellular homeostasis after insecticide application.

Three unigenes encoding ATP-binding cassette (ABC) transporters (contigs ID 1560, 7913, and 2478) were found up-regulated in the S genotype. The ABC transporters belong to a superfamily of proteins involved in extracellular transport of a wide variety of substances, including metabolic products, lipids and xenobiotics [89], [90]. In DDT resistant *Drosophila* strains, differential transcription of ABC transporters has been found, [18]. In the cotton pest *Heliothis virescens*, a mutation in the ABC transporter has been associated with resistance to Bt insecticidal toxins [91]. Therefore, gene sequences for ABC transporters in the S genotype appear to play an important role during insecticide elimination.

Another important group of differentially regulated sequences were unigenes coding for peptidases (contigs ID 256, 254, 3002, 3004, 6594, 7762, 3299, 5268 in S and 3299, 5268 in SR genotypes). In addition, a cysteine-type endopeptidase activity (a feature of cathepsin B) was notably over-represented among the up-expressed sequences in the S genotype. This was consistent with the elevated proteolytic activities observed in insecticide resistant strains of the housefly *Musca domestica* [92], [93] and the maize weevil *Sitophilus zeamais* [94]. In addition, the constitutive over-transcription of genes encoding proteins with peptidase activity has been reported in insecticide resistant insects using a transcriptomic approach [18], [22]. Two different explanations have been proposed for an increased proteolytic activity during insecticide resistance. First, peptidases may be involved in protein degradation to fulfill higher energy demands, which, as afore mentioned, is usually a response to stress [18], [94]. Second, peptidases may play a role during protein biosynthesis or in modification of the enzyme conformation related, for example, with the metabolic machinery required to detoxify insecticides [92], [93].

Finally, four unigenes (contigs ID 3486, 7126 in S genotype, and contigs ID 10027, 4497 in SR genotype) whose putative products correspond to cuticular proteins (CPs) were also found up-regulated. This suggests that the transcriptional plasticity of cuticule proteins may play a central role in insecticide resistance of *M. persicae*, most probably by cuticular thickening or sequestering compounds before entering to the haemolymph. Insecticide resistance through decreased cuticle penetration has been demonstrated in several insect species [7], [95], [96]. A higher constitutive expression of CPs has been reported in insecticide resistant strains of *M. persicae* and *Anopheles gambiae*, and, in the case of *M. persicae,* this was associated with a reduced penetration of the insecticide to the haemolymph [16], [23]. In addition, CPs were found up-regulated in insecticide-resistant strains of the Colorado potato beetle, *Leptinotarsa decemlineata* [8], and in *Aedes aegypti* [21].

### Transcriptomic Responses in the Multiple Resistance Genotype (MR)

The MR genotype, which carries MACE and *kdr* mutations, exhibits a low transcriptional plasticity and can be considered a canalized genotype [97], [98]. This genotype showed a lack of responses, even for the expression of genes encoding enzymes involved in insecticide detoxification (at 20 and 30 hour after insecticide treatment). However, no consistent results were obtained using the qRT-PCR or microarray hibridizations for the guanine nucleotide-binding protein (G-protein, Contig ID 1180), one of the regulated genes we found in this genotype.

### Conclusions

The varied insecticide resistance mechanisms described for *M. persicae* illustrate the complexity of the involved evolutionary responses. Modifications such as single mutations or duplications that could occur in some of the up-regulated genes may be responsible for resistance to high doses of insecticides, accounting for the wide range of potential adaptations to insecticides in this species. On the other hand, asexual reproduction in aphids enables the evolution of “general-purpose” genotypes, because the lack of recombination does not rearrange the co-adaptation among genes, which could be the case in the MR genotype. Our results emphasize the value of microarray studies to search for regulated genes in insects and highlight the many ways these different genotypes can assemble resistant phenotypes in response to the environmental pressures. Further experiments will certainly contribute to develop a more thorough and complete understanding of what genes are regulated in different insect species after the application of different insecticide classes and under different environmental circumstances.

## Materials and Methods

### Aphid Genotypes and Plant Material

Ninety four clonal lineages (genotypes) previously sampled and established in the laboratory were used in this study and genotyped using six microsatellite loci (for details see Castañeda et al. 2011) [99]. Among these, 32 different genotypes were characterized in terms of their insecticide resistance mechanisms. Each genotype was categorized into the following categories: sensitive (S), resistant by a single mutation (SR) and resistant by multiple mutations (MR). Three genotypes, one for each category, were selected for experiments and were maintained in laboratory on leaves of *Capsicum annuum* var. *grossum* (hereafter pepper) in controlled environment (20±1°C and 16L:8D photoperiod). Aphids were synchronized for 24 to 48 hours on three-month old pepper plants before starting experiments.

### Insecticide Resistance Characterization

Constitutive carboxylesterase activity (EST activity) was evaluated in the 32 genotypes reared on pepper using a microplate bioassay [61], with ten independent biological replicates per genotype and three technical replicates per measurement. Broad-sense heritability of enzyme activity was assessed by computing the ratio of inter-clonal variance to phenotypic variance, using the mean squares of a one-way analysis of variance. The presence of insecticide resistance mutations (IRMs) was screened in the 32 genotypes using allelic discrimination based on the quantitative-PCR assays developed by Anstead et al. (2004) for *kdr* (L1014F) and *super-kdr* (M918T) mutations [100], and Anstead et al. (2008) for MACE mutations [101]. See Table S3 for primer and probes sequences. The three genotypes selected for experiments exhibiting low, intermediate and high levels of EST activity.

### Insecticide Tolerance Bioassay

In order to verify the correspondence between IRMs and levels of actual resistance, the three selected genotypes were sprayed with pirimicarb, a carbamate insecticide. The bioassay allowed characterizing the level of tolerance to pirimicarb in the selected genotypes. Toxicity bioassay was performed using the leaf-dip technique [102], with five different insecticide concentrations (ranging between 90 and 1.25 ppm in prepared with acetone plus water) and water as control, with 24 technical replicates per treatment. In brief, pepper leaf-discs were dipped into each insecticide solution and placed in Petri dishes; then, 30 adult wingless aphids were place on each disc. All bioassays were scored at the endpoint, 48 h after treatment, by counting the survivors. The insecticide concentrations lethal to 50% (LC_50_) and 99% (LC_99_) of aphids were calculated using the Probit statistical method [103].

### Insecticide Treatments

Four hundred synchronized adult wingless aphids were placed in groups of 20 individuals on a pepper leaf-disc in Petri dishes containing 2% agar. Then, 10 dishes were sprayed with 1 ml of pirimicarb (20 ppm in acetone plus water) using a Potter-Precision laboratory spray tower (Burkhard) that ensures a homogeneous application [104]. After 24 hours, living aphids were quickly frozen in liquid nitrogen and stored at −70°C until RNA extraction. The other 10 Petri dishes were simultaneously sprayed with water (control). This procedure was performed twice in parallel for the three selected genotypes in order to obtain a minimum of two biological replicates.

### Microarray Hybridization

A microarray containing probes for over 10.000 *M. persicae* unigenes, designed with the Agilent eArray platform (Agilent Technologies) was used [59]. Each slide consisted of eight arrays each containing 60-mers probes (8X60K format).

Total RNA was isolated separately for each experimental condition (genotypes, biological replicates and treatments) from ∼ 40 frozen aphids using the RNeasy Plant Mini Kit (Qiagen, Cat no. 74904). Quantity and quality of RNAs was assessed with a NanoDrop ND-1000 spectrophotometer (NanoDrop® Technologies) and Agilent 2100 Bioanalyzer (Agilent Technologies), respectively. RNA spike-ins (Two-Color RNA Spike-In kit; Agilent) were added to each sample to calibrate the hybridization (the kit was used according to manufacturer’s recommendations only in the case of the S genotype). The Amino Allyl MessageAmp™ II with Cy™3/Cy™5 kit (Ambion) was used to prepare RNA samples for array hybridization. In brief, a reverse transcription from 1.2 mg of total RNA was carried out in each sample using the T7 oligo-dT primer provided in the kit, followed by a second strand cDNA synthesis. Then, double-stranded DNA (dsDNA) was purified with a cDNA filter cartridge and used as template for *in vitro* transcription with the incorporation of aminoallyl modified UTP, which resulted in amplified RNA (aRNA) containing modified UTP. The aRNA was purified with an aRNA filter cartridge and 5 µg were coupled to Cy3 or Cy5 dyes, checking fluorescence with spectrophotometer (NanoDrop® Technologies). Finally, the labeled aRNA was fragmented at 60°C for 30 min and stopped by the addition of 2× GEx Hybridization Buffer HI-RPM as described in the Agilent two-color microarray-based gene expression analysis protocol. All the hybridizations, washed, and scans of microarrays were performed in the Cornell University Life Sciences Core Laboratories Center (http://cores.lifesciences.cornell.edu).

For each genotype, the isolated aRNAs from each condition (insecticide and water) were mixed altogether using opposite dye colors (Cy3 or Cy5 labels). A dye-swap between samples was conducted for each genotype, performing three biological replicates for the S and MR genotypes, and two for the SR genotype. Hence, a total of eight hybridizations were performed (The microarray data sets reported in this paper have been deposited in NCBI’s Gene Expression Omnibus [105] and are accessible through GEO Series accession number GSE37310 (http://www.ncbi.nlm.nih.gov/geo/query/acc.cgi?acc=GSE37310).

Microarray data analysis was performed using the LIMMA library [106] for the statistical package R freely available at: http://www.r-project.org with Bayesian inference using a lineal model [107]. This method allows the joint analysis of all hybridizations performed with each genotype. Normalization within each array was performed using LOESS method, whereas model adjustment was performed using the lmFit function for each genotype [108]. The overall statistical analysis was performed using the eBayes function. We estimated the ratio between fluorescence (insecticide vs. control) in each spot (hereafter Fold-Change or FC); FC values were Log_2_−transformed. Spots showing values within −1>log_2_FC>1 and a P value <0.05, were considered as differentially expressed.

### Annotation and Gene Ontology Analysis

Three datasets containing significantly regulated genes in each genotype were obtained. Given the large amount of information, subsequent analyses were focused only on the up-regulated genes. The Gene Ontology analysis of transcripts was performed using the Blast2GO program [109]. Only BLASTX analysis with a cut-off E-value <1E^-10^ were considered. Then, GO terms were assigned to those sequences using the following parameters: E-Value-Hit-Filter = 1E^−6^; Annotation Cut-Off = 55; GO Weight = 5. This allows the identification of possible roles for each predicted protein, based on three domains of molecular biology: biological processes, molecular functions and cellular components (http://www.geneontology.org) [110], [111].

An enrichment analysis (EA) was also performed in Blast2GO package GOOSIP (Gene Ontology Significance Statistical Interpretation Program, Microdiscovery, Berlin, Germany) [112], [113], in order to compare up-regulated sequences in the S genotype using the entire set of sequences available in the microarray.

### Quantitative Reverse Transcription PCR (RT-qPCR) and Microarray Validation

Two other independent experiments were conducted for each of the three selected genotypes in order to obtain two new biological replicates by treatment. Those experiments were performed as described above, and adding a new level: time after insecticide application (pirimicarb, 20 ppm). Living aphids were recovered 20 and 30 hours after spraying (insecticide or water), and quickly frozen in liquid nitrogen and stored at −70°C until RNA isolation. Seven genes were selected according to significant expression differences (observed in any of the three microarray comparisons) and their putative functions, and expression levels were evaluated by RT-qPCR.

Transcriptional profiles of seven selected genes were validated through RT-qPCR in each of the three selected genotypes. Fresh RNA samples obtained from new biological replicates were used for validation, and included samples isolated 20 and 30 hours after insecticide treatments. Additionally, the transcriptional profiles of three differentially expressed genes were also validated using the same RNA samples used for the microarray experiments in the three genotypes. In the case of new RNA samples, the results were expressed as fold change average obtained in the biological replicates, and in the samples isolated 20 and 30 hours after insecticide application. A correlation coefficient between gene expression measured using microarray and RT-qPCR was calculated using the Spearman’s rho correlation in STATISTICA v.7 [114].

For the new biological replicates, total RNA was isolated from three aphids per genotype using the RNeasy Plant Mini Kit (Qiagen, Cat no. 74904), yielding a range of 100 – 400 ng/µl of RNA (Nanodrop ND-1000, Nanodrop Technologies, USA.). Genomic DNA was removed with DNA-free™ kit (Ambion). Reverse transcription was carried out using the AffinityScript QPCR cDNA Synthesis kit (Agilent) using 1.5 µg of total RNA, which yield about 20 µg of cDNA. Then, the cDNA was diluted to 1∶10, taking 2 µl for PCR reactions. Each PCR reaction mix contained 10 pmol of each primer, 6.25 µl SYBR Green PCR Master Mix (Applied Biosystems) and 0.375 µl of Rox (dilution 1∶500) used as passive reference dye. No template controls (NTC) were included for each PCR to detect external contamination. PCR reactions consisted in 10 min at 95°C, followed by 40 cycles of 15 s at 95°C, 15 s at 57°C and 20 s at 72°C using a Mx3000P QPCR Systems (Stratagene). A dissociation curve was included immediately after each PCR using a ramp of 65–95°C to confirm the absence of nonspecific amplifications and primer dimers. Primers were designed from the sequences of *M. persicae* contigs for seven target genes (GenBank identifiers EC387286, EE261252, EC387215, EE263862, EE262012, EC388935, EE263097) and one endogenous control gene (DW011095), using the package FastPCR (V 5.4.30) and AmplifX (V 1.3.7), and checked in NCBI/Primer-BLAST. Primer sequences, PCR efficiencies and microarray hybridization with up-regulation in the specific gene study are shown in Table S4.

The relative expression ratio of the target gene was computed by relative quantification using the comparative Ct method (Applied Biosystems User Bulletin No. 2 P/N 4303859, 1997) (Livak & Schmittgen, 2001), with the glyceraldehyde-3-phosphate dehydrogenase (GADPH) gene as normalizing endogenous control. Ratios were calculated from a mean normalized expression (MNE), a value that was obtained between biological replicates, as they show a same trend in all cases; MNE value of aphids sprayed with water was used as calibrator. Several studies have validated the use of GAPDH as a reference gene for normalization [115]–[117], and it is one of *M. persicae* most stable endogenous genes in response to insecticides (FC range 0.94 – 0.99, on the microarray presented in this study). In addition, the algorithm NormFinder [118] was used to identify the most stable reference genes among: *GADPH* (DW011095), *cyclophilin-10-like* (EC388830), *ribosomal protein LP0* (DW011949) and *ribosomal protein L7* (DW361765). NormFinder identified GADPH as the most stable expressed gene when all samples were grouped together (stability value of 0.009), as well as when samples were classed into treatments (stability value of 0.016). For each relative expression ratio, we performed a *t-test* between the average and 1, which was used as a reference value for no change in relative expression. The log_2_ of relative expression ratio was calculated to ease the graphical representation.

## Supporting Information

Table S1 **The full list of up-regulated genes, with the log_2_ fold-change values and descriptions based on the closest BLAST hits.**
(XLSX)

Table S2 **Gene expression results for microarray and RT-qPCR methodologies.**
(XLSX)

Table S3 **Primers and probes used for insecticide resistance characterization.**
(XLSX)

Table S4 **Probe name, gene description, primers sequences, PCR efficiency and microarray hybridization with up-regulation for genes study by RT-qPCR.**
(XLSX)
